# Pharmacokinetic Drug-Drug Interaction and Responsible Mechanism between Memantine and Cimetidine

**DOI:** 10.3390/pharmaceutics10030119

**Published:** 2018-08-06

**Authors:** Young A. Choi, Im-Sook Song, Min-Koo Choi

**Affiliations:** 1 College of Pharmacy, Dankook University, Cheon-an 31116, Korea; ayha06@gmail.com; 2 College of Pharmacy and Research Institute of Pharmaceutical Sciences, Kyungpook National University, Daegu 41566, Korea; isssong@knu.ac.kr

**Keywords:** mematine, drug interaction, liquid chromatography-tandem mass spectrometry, pharmacokinetics

## Abstract

A sensitive and simple chromatography-tandem mass spectrometry (LC-MS/MS) method was developed to evaluate memantine in rat plasma. Memantine and propranolol (internal standard) in rat plasma was extracted using a methanol precipitation method. The standard curve value was 0.2–1000 ng/mL and selectivity, linearity, inter-day and intra-day accuracy and precision were within acceptance criteria. Using this validated method, drug-drug interactions between memantine and cimetidine was measured following co-administration of memantine and cimetidine intravenously and orally. Plasma exposure of memantine was increased by 1.6- and 3.0-fold by co-medication with cimetidine intravenously and orally, respectively. It suggested that the drug interaction occurred during the gut absorption process, which was consistent with the results showing that the intestinal permeability of memantine in the presence of cimetidine was 3.2-fold greater than that of memantine alone. Inhibition of cimetidine on hepatic elimination of memantine rather than renal excretion was also attributed to the drug-drug interaction between memantine and cimetidine, which explained the decreased clearance of memantine by co-medication with cimetidine. In conclusion, the newly developed simple and sensitive LC-MS/MS analytical method was applied to investigate the pharmacokinetic drug-drug interactions of memantine. Plasma exposure of memantine by co-administration with cimetidine was increased because of its enhanced intestinal permeability and the decreased metabolic activity of memantine.

## 1. Introduction

Alzheimer’s disease is among the most common dementia and is progressed by memory loss, confusion, impaired judgement and disorientation [1]. Currently drugs that can completely cure Alzheimer’s disease are not available [2]. Accessible medications focus on symptomatic treatment and only reduce disease progression [2]. Memantine (Namenda^®^) is *N*-methyl-d-aspartate antagonist in the brain [3] which has been approved by US Food and Drug Administration (FDA) to treat moderate to severe dementia. Memantine also inhibits the neurodegenerative effects of glutamate [4] and protects neuron cells [5]. Based on this mode of action, memantine showed promising clinical and preclinical effects on several psychiatric disorders such as bipolar disorder, depression, schizophrenia and anxiety disorders as mono or add-on therapy [6]. Therefore, memantine has been prescribed alone or in combination with other cognitive drugs such as donepezil (Aricept^®^), rivastigmine (Exelon^®^) and galantamine (Razadyne^®^) as well as anti-psychotic drugs [6,7]. It has been reported that no drug-drug interaction between memantine and donepezil, rivastigmine and galantamine was observed [8,9,10]. However, it may interact with other *N*-methyl-d-aspartate receptor ligands such as dextromethorphan, ketamine and phencyclidine and anti-psychotic drugs such as amantadine and dopamine antagonists [6]. Drug-drug interaction may change the pharmacokinetic features and therapeutic response of memantine. In addition, Dizziness and vomiting are common adverse events of memantine, which may be associated with the high plasma concentrations of memantine [5].

Because of its pharmacokinetic-pharmacodynamic relationship, the therapeutic drug monitoring of Alzheimer’s disease drugs as well as anti-psychotic drugs is increasingly necessary for studying individual variations, metabolic and kinetic alterations in some patients, responsible mechanisms for decreasing effectiveness and to make a decision to adherence or changing existed therapy [11]. Moreover, to investigate the mechanisms responsible for the possible interaction between memantine and other drugs which may cause the high plasma concentration of memantine, it is necessary to establish an accurate, sensitive and simple bioanalysis method for the detection of memantine in diverse matrices.

Therefore, the aim of this study was to develop and validate a sensitive and simple liquid chromatography tandem mass spectrometry (LC-MS/MS) method for measuring memantine focusing on lowering the required plasma volume and simplifying the overall process. The method was fully validated according to the FDA Guideline for Bioanalytical Method for its linearity, selectivity, accuracy, precision, recovery, stability and matrix effects [12].

To examine application of this analytical method, we investigated pharmacokinetic drug–drug interactions between memantine and cimetidine. To understand the underlying mechanisms of the drug-drug interaction between memantine and cimetidine, we administered memantine with or without cimetidine intravenously as well as orally. Cimetidine was selected because it inhibits cytochrome P 450 (Cyp)-mediated metabolism and the organic cation transporter (Oct)-mediated transport process [13,14]. In addition to Cyp and Oct inhibition, cimetidine, an H2 receptor antagonist, is used to treat peptic ulcers and is the most prescribed over-the-counter drug that is easily accessed by patients [15,16]. Cimetidine is well-absorbed from the gut through the paracellular transport and mainly excreted via the renal route in rats (70% of oral dose was eliminated via the renal route for 72 h) with limited metabolism and low plasma protein binding (approximately 20%) [17,18], suggesting that a high plasma concentration of cimetidine is maintained. Thus, the plausibility of concomitant administration of memantine and cimetidine and drug-drug interaction between memantine and cimetidine seems to be high.

## 2. Materials and Methods

### 2.1. Materials

Memantine, cimetidine and propranolol was purchased from Sigma-Aldrich (St. Louis, MO, USA). Methanol and water was of HPLC grade (Burdick & Jackson Korea, Seoul, Korea). All other reagents and solvents were of reagent or analytical grade.

### 2.2. Animals

Male Sprague-Dawley rats (7–8 weeks, 220−250 g) were purchased from Samtako (Osan, Kyunggi-do, Korea). They were acclimatized to the circumstances for 5 days with water and food ad libitum. Rats were provided a cereal-based maintenance diet, altromin 1320 pellet formula (Altromin, Lage, Germany). Energy density of this diet is about 3227 kcal/kg consisting of 11% of fat, 24% of protein and 65% of carbohydrates. All rats were housed in light (light: 07:00–19:00, dark: 19:00–07:00), temperature (22 ± 2 °C) and humidity (55 ± 5%)-controlled room.

All animal procedures were approved by the Animal Care and Use Committee of Kyungpook National University (2015-0098) and conducted in accordance with the National Institutes of Health guidance for the care and the use of laboratory animals.

### 2.3. LC-MS/MS Analysis of Memantine

Memantine in each sample was determined by an Agilent 6430 Triple Quadrupole LC-MS/MS system (Agilent, Wilmington, DE, USA). The isocratic mobile phase consisting a mixture of water and methanol (15:85, *v*/*v*) containing 0.1% formic acid was used at a flow rate of 0.20 mL/min to elute memantine and propranolol peak from the rat plasma matrix. The separation was performed on a Polar RP column (150 × 4.6 mm, 5 μm particle size; Pheonomenex, Torrance, CA, USA).

Mass transition was monitored using multiple reaction monitoring (MRM) mode at *m*/*z* 180.1 → 163.1 for memantine and *m*/*z* 260.0 → 116.0 for propranolol (internal standard, IS) in positive ion mode with a collision energy of 10–15 eV. The analytical data were quantified using mass Hunter (version B.06.00, Agilent, Wilmington, DE, USA).

### 2.4. Method Validation

#### 2.4.1. Selectivity

The representative MRM chromatograms of the standard lower limit of quantification (LLoQ) concentrations of memantine or propranolol (IS) and plasma samples obtained from a rat 10 h after the oral administration of memantine (5 mg/kg) were compared with those of blank plasma [19].

#### 2.4.2. Linearity of Standard Curves

The calibration curve (0.2, 0.5, 2, 10, 50, 200, 1000 ng/mL) was prepared using an internal standard method. Aliquots of standard curve samples (50 μL) were added to 250 μL of methanol containing 20 ng/mL of propranolol (IS). After vortex-mixing and centrifugation for 10 min at 13,200 rpm, an aliquot (2 μL) of the supernatant was injected into the LC-MS/MS system. Linearity of the calibration standard was calculated from the peak response ratio of memantine to IS with the weight adjusted method (1/*x*^2^).

#### 2.4.3. Precisions and Accuracy

The precision and accuracy of inter- and intra-day assays were assessed from five measurements of the quality control (QC) samples (0.6, 20, 500 ng/mL) of memantine. Precision was evaluated from the relative standard deviation (RSD) of five measurements. The accuracy of inter-day and intra-day assays was calculated by dividing the measured QC concentration by the spiked QC concentration. Analytical sample preparations were identical during the validation process.

#### 2.4.4. Matrix Effect and Recovery

Matrix effects were monitored by dividing the peak areas of QC samples in water by those in blank plasma [20]. Recovery was calculated by comparing the peak response of QC in the post-extraction spiked samples with those of the pre-extraction spiked samples [20].

#### 2.4.5. Stability

The stability of memantine and IS in the rat plasma was tested from the low and high QC samples exposed to different conditions [19]. Short-term stability was calculated by comparing QC samples that were stored for 12 h at 25 °C with the untreated QC samples. The freeze-thaw stability was analyzed by comparing QC samples that underwent three freeze–thaw cycles (−80 °C to 25 °C as one cycle) with the untreated QC samples. Post-preparative stability was evaluated by analyzing the extracted QC samples maintained in the autosampler at 6 °C for 24 h compared with the untreated QC samples [19].

### 2.5. Pharmacokinetic (PK) Interaction Study

Before oral administration or rat jejunum resection, the rats were fasted for 12 h but water ad libitum. On the day of study, the femoral arteries and femoral veins of rats were cannulated with PE50 tubing (Jungdo, Seoul, Korea) under anesthesia with isoflurane (30 mmol/kg).

Cimetidine solution (10 mg/kg/mL in water, four rats) or vehicle solution (1 mL/kg of water, four rats) was injected intravenously to rats at 10 Am and, 5 min later, memantine solution (3 mg/kg/mL in water) was injected intravenously to the same rat. Blood samples (250 μL) were collected from the femoral artery at 0, 0, 0.25, 0.5, 0.75, 1, 1.5, 2, 3, 6, 9, 24 h after intravenous bolus injection of memantine and/or cimetidine. Blood samples were centrifuged for 10 min at 13,200 rpm and aliquots of plasma samples (50 μL) were stored at −80 °C until the analysis. Urine samples were collected for 24 h and pooled urine samples were weighed. 50 μL aliquot of urine samples was stored at −80 °C until the analysis.

Similarly, cimetidine solution (50 mg/kg, dissolved in 2 mL water, four rats) or vehicle solution (2 mL/kg of water, four rats) was administered orally to rats at 10 Am and 30 min later, memantine solution (5 mg/kg, dissolved in 2 mL water) was administered orally to the same rat. Blood samples (250 μL) were collected from the femoral artery at 0, 0.083, 0.25, 0.5, 1, 2, 3, 6, 9, 24 h after oral administration of memantine and/or cimetidine. Blood samples were centrifuged for 10 min at 13,200 rpm and aliquots of plasma samples (50 μL) were stored at −80 °C until the analysis.

Memantine concentrations in plasma and urine samples were analyzed using a LC-MS/MS system.

### 2.6. Intestinal Permeability Test

Apparent permeability (P_app_) of memantine in the presence or absence of cimetidine was determined with slight modification of a method of Kwon et al. [21]. Briefly, the rat jejunum segments mounted on the inserts of the Ussing chambers were acclimatized with Hank’s balanced salt solution (HBSS) for 15 min. Carbogen gas (5% CO_2_/ 95% O_2_) was bubbled into the Ussing chambers at a rate of 150 drops/min during the experiment for the jejunum segment viability. The experiments started with changing the HBSS with preheated HBSS containing memantine (0.5 mg/mL) with or without cimetidine (5 mg/mL) to the apical (A) side of jejunum. Aliquots (400 μL) of HBSS in the basal side (B) were withdrawn at 0, 30, 60, 90 and 120 min and compensated with an equal volume of fresh, preheated HBSS. Aliquots of 50 μL of samples were analyzed using a LC-MS/MS system applied to the same sample preparation method.

For system feasibility, P_app_ of caffeine, propranolol, ofloxacin and atenolol were monitored as marker compounds for high, moderate and low permeable drugs. P_app_ value of caffeine and propranolol, positive controls of high permeability, was 36.8 × 10^−6^ and 23.7 × 10^−6^ cm/s, respectively, which is consistent with the findings of other studies [22]. P_app_ value of ofloxacin and atenolol, a positive control of moderate and poor permeability, respectively, was 4.5 × 10^−6^ and 0.04 × 10^−6^ cm/s, respectively, which corroborates with the findings of other studies [23].

### 2.7. Microsomal Stability

The metabolic stability of memantine (1 μM) in the presence or absence or cimetidine (0, 2 and 20 μM) in the rat liver microsomes was measured as previously described [24]. Briefly, memantine solution (1 μM) in 100 mM potassium phosphate buffer (pH 7.4) containing 0.5 mg of liver microsomes (from Sprague-Dawley rats; Corning, Tewksbury, MA, USA) and an NADPH-generating system incubated at 37 °C for 0, 15, 30, 45 and 60 min in a shaking water bath. The reaction was quenched by adding ice-cold methanol containing 20 ng/mL of propranolol (250 μL). After undergoing the sample preparation procedure, memantine concentration was measured.

We also measured the metabolic stability of 1 μM metformin and 1 μM propranolol by the same procedure to ensure system feasibility [24]. The percent of remaining metformin and propranolol after 60 min incubation in the rat liver microsomes was 63.4% and 12.2%, respectively.

### 2.8. Data Analysis and Statistics

PK parameters were calculated using the WinNonlin 2.0 (Pharsight Co., Certara, NJ, USA) and the data are expressed as the means ± standard deviation (SD). 

A value of *p* < 0.05 was determined to be statistically significant, using a Mann Whitney test between the two means for unpaired data.

## 3. Results

### 3.1. Validation of Analytical Method

#### 3.1.1. Selectivity

**Figure 1:**
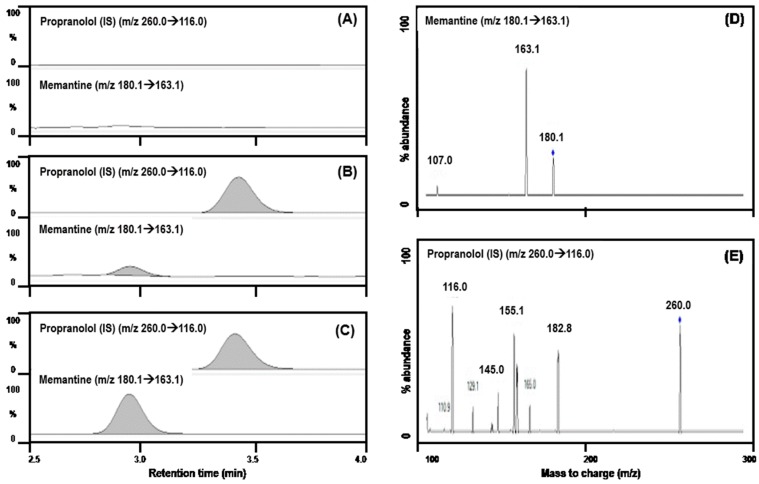
Representative MS/MS chromatograms of memantine and IS in rat (**A**) double blank plasma, (**B**) blank plasma spiked with memantine at lower limit of quantification (LLoQ; 0.2 ng/mL) and propranolol and (**C**) plasma samples following single oral administration of memantine is shown in the left panel. Product ion spectra of memantine (**D**) and propranolol (**E**) are shown in the right panel.

Representative MS/MS chromatograms memantine and IS (Figure 1) showed that the memantine and IS peaks were well-separated with no interfering peaks at the respective retention times. The selectivity of the analytes was confirmed in six different rat blank plasma and rat plasma samples obtained after memantine administration. The retention times of memantine and IS were 3.62 and 3.14 min, respectively and the total run time was 4.0 min.

#### 3.1.2. Precision and Accuracy

The precision and accuracy of intra-day and inter-day assays are shown in Table 1. For inter-day validation, the precision was in the range of 8.10 to 11.4%. The accuracy ranged from 101.2 to 108.6%. In intra-day validation, the precision and accuracy ranged from 1.68 to 14.1% and 109.9 to 113.8%, respectively (Table 1).

#### 3.1.3. Matrix Effect and Recovery

The mean recoveries of memantine in the low, medium and high QC samples ranged from 87.5 to 91.2% with an RSD of less than 3.70%. The matrix effects ranged from 91.1 to 108.2% with an RSD of less than 12.6%. These results indicate that no significant interference occurred during the ionization and methanol precipitation process. The matrix effect of the IS at 20 ng/mL was 102.9% and the recovery of IS was 87.7% (Table 2).

#### 3.1.4. Stability

The stabilities of memantine and propranolol are summarized in Table 3. Low and high QC samples were stable after being exposed to three freeze-thaw cycles and stable for at least 12 h at 25 °C. Post-preparative samples were stable after storage at 24 h in the autosampler after sample preparation.

### 3.2. PK Interaction of Memantine and Cimetidine in Rats

**Figure 2:**
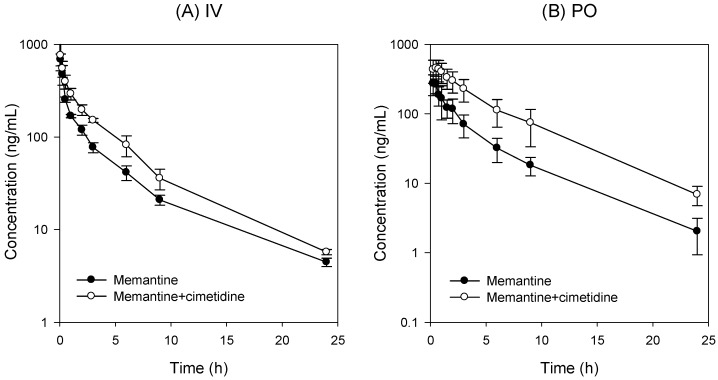
Plasma concentration-time profile of memantine after single intravenous (IV; **A**) and oral (PO; **B**) administration of memantine (3 and 5 mg/kg, respectively) in the presence (○) or absence (●) of cimetidine (10 and 50 mg/kg, respectively) in rats. Data points are the means ± SD of four rats.

The PK profiles of memantine following intravenous injection (3 mg/kg) in the presence or absence of cimetidine (10 mg/kg) are shown in Figure 2A and PK profiles of memantine following oral administration (5 mg/kg) in the presence or absence of cimetidine (50 mg/kg) are shown in Figure 2B. Their related PK parameters are presented in Table 4. The pharmacokinetic parameters of memantine were comparable to those reported previously [3,7,25].

There was no significant difference in the C_max_, t_1/2_ and V_d,ss_ values of memantine between two groups (memantine alone vs. memantine + cimetidine). However, the AUC value of memantine + cimetidine was increased by 1.6-fold compared to that of memantine alone and, consequently, the CL of memantine decreased by co-injection with cimetidine.

Interestingly, the drug–drug interaction between memantine and cimetidine administered orally was greater than that of intravenous injection. For example, the AUC value of memantine + cimetidine was increased by 3.0-fold compared to that of memantine alone without changing the T_max_ and t_1/2_ values. These results suggest that a major drug–drug interaction between memantine and cimetidine occurred during the absorption process. Moreover, systemic clearance (CL and CL/F) of memantine was decreased by co-administration of memantine and cimetidine by both the oral and intravenous routes, suggesting that memantine elimination is affected by the presence of cimetidine.

To investigate the underlying mechanism of the drug interaction in the absorption process, we investigated the effect of cimetidine on the intestinal permeability of memantine.

For the elimination process, we investigated the changes in metabolic stability in the rat liver microsomes because renal elimination (CL_renal_) was not significantly altered by the presence of cimetidine (Table 4) and, therefore, non-renal clearance of memantine (calculated by subtracting CL_renal_ from CL) was decreased by the presence of cimetidine.

### 3.3. Effect of Cimetidine on the Intestinal Permeability of Memantine

To mimic the intestinal situation that occurred when memantine and cimetidine were concomitantly administered to rats via oral route, the concentrations of memantine and cimetidine were determined based on the oral dose of these drugs used in pharmacokinetic analysis and fluid volume of the stomach. For example, memantine and cimetidine were administered at doses of 5 and 50 mg/kg (dissolved in 2 mL/kg water), respectively, to rats with stomach fluid volumes of 3.4–4.6 mL [26], which resulted in 8–10-fold dilution.

**Figure 3:**
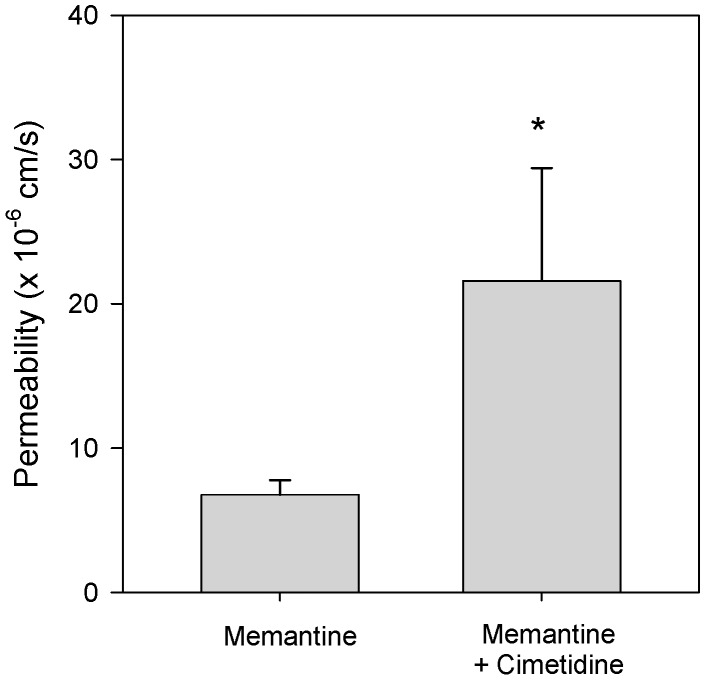
The apparent permeability (P_app_) of memantine (0.5 mg/mL) in the absence or presence of cimetidine (5 mg/mL) was measured in rat jejunum using the Ussing system. Bar represents the mean ± SD of three independent experiments. * *p* < 0.05 compared with the memantine group.

The apparent permeability of memantine (0.5 mg/mL) in the presence of cimetidine (5 mg/mL) was significantly increased compared to that of memantine alone (Figure 3).

### 3.4. Effect of Cimetidine on the Metabolic Stability of Memantine

**Figure 4:**
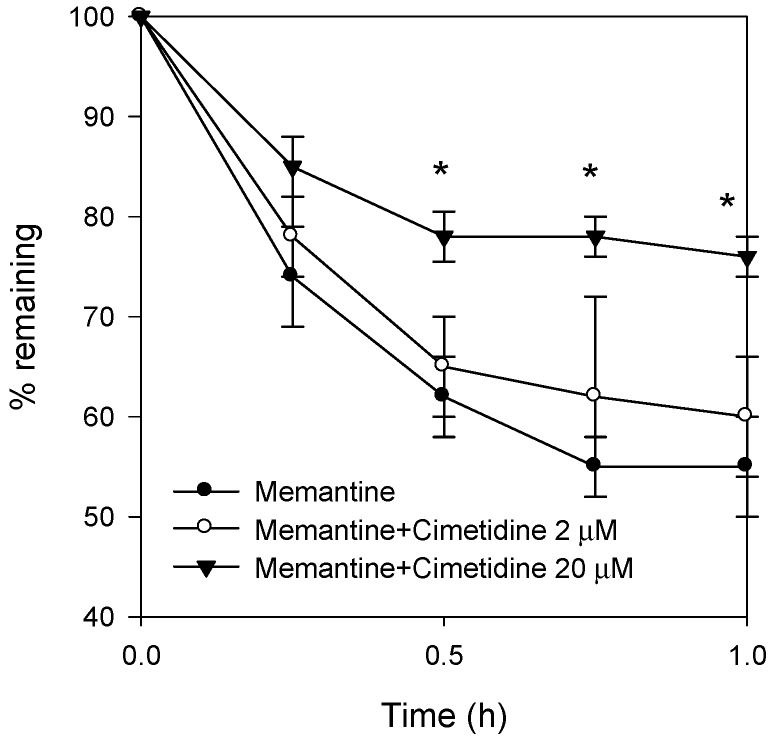
The effect of cimetidine on the metabolic stability of memantine with rat liver microsomes. Memantine (1 μM) in the absence or presence of cimetidine (0, 2 and 20 μM) was incubated with 0.5 mg rat liver microsomes for 60 min at 37 °C. Data represents the mean ± SD of three independent experiments. * *p* < 0.05 compared with the memantine group.

Approximately 45% of memantine was degraded after 1 h incubation of memantine in the rat liver microsomes (Figure 4), which is consistent with the results of a previous study showing that memantine exhibits rat-specific Cyp-dependent moderate to high intrinsic clearance [3]. Adding 2 and 20 μM cimetidine and 1 μM memantine to the rat liver microsomes showed that the metabolic rate of memantine decreased with increasing concentrations of cimetidine (Figure 4).

## 4. Discussion

The newly developed analytical method for memantine using an LC-MS/MS system showed comparable sensitivity (i.e., LLoQ 0.2 ng/mL) as previously published methods (i.e., LLoQ 0.1–1 ng/mL) despite the use of a small volume (50 μL) of plasma samples compared with previous methods [7,27,28,29,30,31]. Additionally, previously established methods used solid-phase extraction or liquid-liquid extraction which requires costly reagents and large volume of plasma samples (100–1000 μL) [27,28,29,30,31]. Noetzli et al. applied the protein-precipitation method and sample preparations were processed by evaporation and reconstitution [29]. However, we used a protein-precipitation method involving methanol containing an internal standard rather than previously described solid-phase extraction or liquid–liquid extraction methods and then directly injected an aliquot of the supernatant after the centrifugation of protein-precipitated plasma samples. Finally, we developed and fully validated a rapid, simple and sensitive analytical method for memantine in biological samples with total run time of 4.0 min. This method can easily be applied in the bioanalysis and pharmacokinetic study of memantine in small experimental animals. Moreover, following appropriate validation, the present method can be extended to determine the routine drug monitoring of memantine in human plasma and thus applied to clinical pharmacokinetic studies.

Memantine showed non-linear pharmacokinetic properties in a single oral dose range of 1–10 mg/kg. Beconi et al. reported that the active process mediated by rat-specific Cyp and active tubular secretion modulated the non-linear pharmacokinetics of memantine [3]. Cimetidine (as a perpetrator) increased the plasma concentration of the victim drug by inhibiting Cyp-mediated metabolism [14] and organic cation-mediated renal tubular secretion [13,14].

Based on these pharmacokinetic properties, the pharmacokinetics drug-drug interaction between memantine and cimetidine could be summarized as follows: (1) the AUC increase of memantine by co-administration with cimetidine was much greater following oral administration of memantine and cimetidine than that following intravenous injection. This suggests that the drug interaction was dominantly induced during the gut absorption process, which was supported by the increased permeability of memantine following addition of cimetidine (Figure 3). Although the intestinal absorption process of memantine has not been fully postulated, memantine absorption increased from 36.3% to 84.9% when oral dose increased from 1 mg/kg to 10 mg/kg, suggesting the presence of active process [3]. The involvement of pH-dependent transport mechanism via Octn1 has been reported in the distribution of memantine [32,33] and cimetidine could efficiently increase the influx of memantine through the inhibition of Octn1 by the inhibition of inwardly directed proton gradient and increase of gut pH [32,33]. (2) The AUC increase of memantine by co-administration of memantine and cimetidine intravenously may be attributed to the decreased clearance of memantine because of the decreased metabolic activity of memantine in the presence of cimetidine (Figure 4). This was also consistent with the previous reports that the intrinsic clearance calculated as moderate to high for rats, in which rat Cyp2c11 is responsible for the memantine metabolism [3] and cimetidine could inhibit hepatic Cyp2C6 and Cyp2C11 enzymes [34]. (3) Inhibition of organic cation-mediated renal tubular secretion of memantine by co-administration with cimetidine was not observed in this study, possibly because the renal clearance of memantine was not significantly decreased by the presence of cimetidine (Table 4). In conclusion, taken together, the pharmacokinetic interaction between memantine and cimetidine in rats was occurred not only at hepatic metabolism but also at intestinal membrane.

The clinical drug-drug between memantine and cimetidine was not investigated yet. However, approximately 48% of memantine was excreted as its unchanged form via renal route in human, in which multidrug and toxin extrusion protein (MATE) 1 was known to be involved [6,35]. The remaining 50% of memantine is metabolized into N-glucuronide conjugate, 6-hydroxy memantine and 1-nitroso deaminated memantine [6]. Since cimetidine acts as inhibitors of MATE1 and CYP enzymes [14,34], possibility of clinical drug-drug interaction between memantine and cimetidine should be carefully considered at high dose administration of cimetidine with memantine.

## Figures and Tables

**Table 1:** Intra-day and inter-day precision and accuracy of memantine.

Memantine	Spiked (ng/mL)	Inter-day			Intra-day		
		Measured (ng/mL)	Accuracy (%)	Precision (%)	Measured (ng/mL)	Accuracy (%)	Precision (%)
Low QC	0.6	0.62 ± 0.05	101.2	8.10	0.68 ± 0.10	113.8	14.1
Medium QC	20	20.6 ± 2.13	108.6	10.3	21.98 ± 0.37	109.9	1.68
High QC	500	497.6 ± 56.9	104.2	11.4	555.6 ± 23.5	111.2	4.23

Data represent the means ± SD of five measurements.

**Table 2:** Matrix effect and recovery of memantine.

Compounds		Spiked (ng/mL)	Recovery (%)	Matrix Effect (%)
Propranolol	IS	20	87.7 ± 1.96 (2.2)	102.9 ± 1.20 (1.2)
Memantine	Low QC	0.6	90.7 ± 3.11 (3.4)	108.2 ± 13.7 (12.6)
	Medium QC	20	91.2 ± 3.38 (3.7)	92.7 ± 5.43 (5.9)
	High QC	500	87.5 ± 3.18 (3.6)	91.1 ± 7.55 (8.3)

Data represent the means ± SD of five measurements.

**Table 3:** Stability of memantine.

Spiked (ng/mL)	Measured (ng/mL)	Accuracy (%)	Precision (%)
Bench-top stability for 12 h in plasma			
Low QC (0.6)	0.67 ± 0.10	112.2	14.9
High QC (500)	532.7 ± 27.6	106.6	5.2
Three freeze-thaw cycles			
Low QC (0.6)	0.51 ± 0.05	85.1	10.7
High QC (500)	539.3 ± 16.4	107.9	3.1
Post treatment stability for 24 h			
Low QC (0.6)	0.59 ± 0.04	98.4	7.6
High QC (500)	540.1 ± 13.7	108	2.5

Data represent the means ± SD of five measurements.

**Table 4:** PK parameters of memantine after single intravenous (IV) and oral (PO) administration of memantine in the presence or absence of cimetidine in rats.

PK Parameters		IV		PO	
		Memantine (3 mg/kg)	Memantine (3 mg/kg) + Cimetidine (10 mg/kg)	Memantine (5 mg/kg)	Memantine (5 mg/kg) + Cimetidine (50 mg/kg)
C_max_	μg/mL	686.84 ± 100.75	759.04 ± 240.18	293.45 ± 90.90	486.78 ± 140.75 *
T_max_	h			0.35 ± 0.14	0.50 ± 0.18
AUC_24h_	μg·h/mL	1089.58 ± 116.55	1724.52 ± 241.36 *	811.38 ± 238.01	2392.69 ± 950.06 *
AUC_∞_	μg·h/mL	1121.98 ± 116.74	1765.56 ± 237.78 *	825.60 ± 241.92	2439.28 ± 951.72 *
t_1/2_	h	5.06 ± 0.38	4.98 ± 0.59	4.51 ± 0.92	4.70 ± 0.75
V_d,ss_	L/kg	19.70 ± 3.32	12.50 ± 3.16		
CL	mL/min/kg	44.87 ± 4.40	28.67 ± 3.92 *		
CL/F	mL/min/kg			107.63 ± 28.83	39.06 ± 16.00 *
CL_renal_	mL/min/kg	12.88 ± 3.89	10.64 ± 5.73		

Data were expressed as mean ± SD from four rats per group. *: *p* < 0.05 compared with the memantine group. C_max_: maximum plasma concentration; T_max_: time to reach C_max_. AUC_24h_ or AUC_∞_: Area under plasma concentration-time curve from zero to 24 h or infinity. t_1/2_: elimination half-life; V_d,ss_: volume of distribution at steady-state CL or CL/F: systemic clearance; CL_renal_: renal clearance.
